# Application of osteoinductive calcium phosphate ceramics in children’s endoscopic neurosurgery: report of five cases

**DOI:** 10.1093/rb/rby011

**Published:** 2018-05-25

**Authors:** Jia Wei, Hufei Qian, Yu Liu, Jiangang Liu, Rui Zhao, Xiao Yang, Xiangdong Zhu, Ruoping Chen, Xingdong Zhang

**Affiliations:** 1 Department of Neurosurgery, Shanghai Children’s Hospital, Shanghai Jiao Tong University, Shanghai, China and; 2 National Engineering Research Center for Biomaterials, Sichuan University, Chengdu, China

**Keywords:** neuroendoscopy, skull holes, CSF leaking, osteoinductive CaP ceramics, bone healing

## Abstract

This work aimed at investigating the possibility and effectiveness of osteoinductive calcium phosphate (CaP) ceramics to close the drilled skull holes and prevent the postoperative cerebrospinal fluid (CSF) leaking in children’s endoscopic neurosurgery. Five children patients (four boys and one girl, 3- to 8-years old) underwent the surgery, in which the endoscopic third ventriculostomy (ETV) was operated in four cases of hydrocephalus, and biopsy and ETV were both performed in one case of pineal tumor. The drilled skull holes were filled with the commercial osteoinductive CaP ceramics. The patients were followed up by CT scan at 1, 7 days, 3 and 6 months postoperatively. All the five cases were successful, and the holes were closed well after filled with the ceramics. The follow-up survey showed that no CSF leaking or rejection reaction was found. The CT scan indicated that the drilled holes began healing at 7 days postoperatively, and a relatively complete healing happened at 6 months postoperatively. The excellent ability of the CaP ceramics to induce bone regeneration was also confirmed by repairing the skull defects in a monkey model. The results of μ-CT and histological analysis showed that a bony structure with irregular array occurred at the defect area, and the newly formed bone volume density reached 65.7%. In conclusion, the osteoinductive CaP ceramics could be an ideal material to treat the drilled skull holes in children’s endoscopic neurosurgery and prevent CSF leaking afterwards. However, further investigation with more cases and longer follow-up was required to evaluate the clinical effect.

## Introduction

Neuroendoscopy is one of the most important surgical managements in minimally invasive neurosurgery [1, 2]. With technological advance, this surgical technique has been adapted to children [3–7]. However, it is undefined how to deal with the drilled skull holes due to the endoscopic surgery, and inappropriate treatment may lead to cerebrospinal fluid (CSF) leaking, especially in infants and young children [8–10]. So, the management of CSF leaking is very difficult after the neuroendoscopic surgery. Due to the existence of the cortex track communicating with ventricle, as well as the limited size of the drilled skull hole, the hard mask is hard to be sutured, leading to the permanent existence of the CSF leaking. In that case, it is imperative to repair the hard mask again by second operation. Otherwise the persistent CSF leaking could induce the incision or even the intracranial infection.

In order to prevent the CSF leaking, some surgeons adjusted the position of the drilled skull holes to avoid the holes directly facing the incision. Besides the special surgical method, a bio-inert sealing agent, i.e. bone wax, was used to close the skull holes after the operation [11]. However, the foreign body reaction resulted from the bone wax could lead to the delayed healing of the scalp wound in the children patients. Moreover, the weak biocompatibility of bone wax lead to difficulty in integrating with the host bone, the frequently occurred drifting of the material would result in the failure of the sealing.

Calcium phosphate (CaP) ceramics have the similar inorganic components of the natural bone and have exhibited excellent biocompatibility, osteoconductivity and osteoinductivity when used as artificial bone substitutes [12, 13]. Generally, the highly interconnected porous structure is essential for the osteoinductivity of CaP ceramics [14–16]. It is known that the highly porous scaffolds should have good air permeability. Therefore, when used for repairing skull defects, the porous CaP ceramic could decrease the intracranial pressure. However, the water permeability of the porous ceramic is dependent on its permeability and the inner and outer pressure difference. With better bone repair ability, the porous CaP ceramic with excellent osteoinductivity could have the potential to inhibit the CSK leaking after neuroendoscopic surgery. To verify this assumption, a commercial osteoinductive CaP ceramic was used in this study to seal the drilled holes during the endoscopic surgery, and its sealing effect was remarkable in the short-term follow-up.

## Materials and methods

### Materials

Commercial osteoinductive CaP ceramics (Trade name: Osteoinductive artificial bone) were supplied by Engineering Research Center in Biomaterials, Sichuan University, China. This ceramic was composed of hydroxyapatite (HA) and β-tricalcium phosphate (β-TCP), and the ratio of HA to β-TCP was about 20/80. The sterilized samples (γ-ray irradiation) with the size of Φ 10 × 5 mm^3^ were used in this study. The morphology, porous structure and permeability were analyzed by field emission scanning microscopy (S4800, Hitachi, Japan) and automatic mercury porosimeter (AutoPore IV 9500, Micromeritics, America), respectively.

### Animal experimental study

To evaluate the effect of the CaP ceramics on the skull defect repair, a preliminary animal experiment was performed in WestChina-Frontier PharmaTech Co., Ltd. (WCFP)/National Chengdu Center for Safety Evaluation of Drugs (NCCSED). The experimental scheme was checked and approved by Institutional Animal Care and Use Committee (IACUC).

An adult rhesus monkey (male, 5.4 Kg) was used in this study. After tracheal intubation anesthesia, the head hair of the animal was shaved by an electric razor, and the head was sterilized by iodine. A 3-cm incision parallel to the mid-line was made in the right of the head, and then a skull hole of 1.0 cm in diameter was drilled by a hand trephine. The CaP ceramic sample was reshaped appropriately and fitted to the skull hole, followed by spraying a certain amount of normal saline to wet the material and make it adhere to the surrounding bone. Finally, each layer of the scalp was tightly sutured. The monkey was sacrificed at 6 months postoperatively. The bone specimen containing the implant was retrieved and then fixed in 4% paraformaldehyde solution for 7 days for the subsequent analysis.

High-resolution micro-computed tomography imaging (µ-CT, SCANCO VivaCT80, Switzerland) was used to assess new bone formation within the implant. Scanning was performed at 70 kV and 114 µA in high-resolution mode, 2048 rescontruction pixels and 200 ms integration time. Differences in voxel size (e.g. 10–20 μm) have little influence on the evaluation of trabeculae structures in large animal models with relatively high thickness (i.e. 100–200 μm) [1]. Therefore, the 2D gray-scale images of the skull of an adult monkey were scanned at an isotropic voxel size of 15 μm. The obtained radiographic images were then stored in DICOM format and reconstructed using MIMICS 17.0 (Materialise, Leuven, Belgium). A threshold (value = 1885) was used to exclude the interference of non-mineralized tissue and a higher threshold (value = 6509) was used to distinguish the material from the mineralized bone tissue. After thresholding, the ratio of the calculated new bone volume (BV) to the total volume (TV) at the defect site was determined as the bone volume fraction (BV/TV).

After μ-CT analysis, the fixed specimen by 4% paraformaldehyde solution was decalcified in 10% ethylene diamine tetraacetic acid (EDTA) solution, dehydrated in ascending concentrations of alcohols from 75 to 100%, and embedded in paraffin. The sections were cut, ground and polished to a final thickness of ∼5 μm, and then transferred onto 3-aminopropyltriethoxysilane-coated glass slides. Finally, the sections were subjected to the hematoxylin and eosin (H&E) staining for histological observation.

### Clinical study

The clinical research was conducted in the Shanghai Children’s Hospital affiliated to Shanghai Jiao Tong University, China. There were five children in this group, four boys and one girl, aged from 3- to 8-years old, four cases of hydrocephalus (including obstructive and communicating hydrocephalus), and one case of pineal tumor with obstructive hydrocephalus.

**Figure 1.**
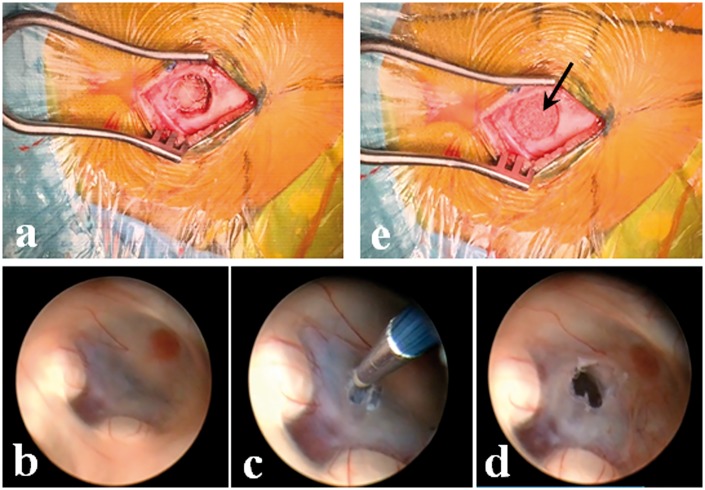
The Surgical procedure in children’s endoscopic neurosurgery

All patients were operated with tracheal intubation anesthesia in a supine position and Mayfield head holder. A 3.0-cm longitudinal incision was made about 1.0 cm in front of the right coronal suture and 3.0 cm next to the mid-line, and then a frontal skull hole of about 1.0 cm in diameter was drilled (Fig. 1a).

All the five cases were treated with endoscopic third ventriculostomy (ETV). Endoscope was firstly punctured into the lateral ventricle and entered into the third ventricle through the interventricular foramen (Fig. 1b), and then, a small hole was formed at the weakest part of the trigonum in front of both mammillary bodies by bipolar electrocoagulation burning (Fig. 1c). After that, a balloon catheter for dilation was inserted into the hole to expand the fistula to about 0.5 cm in diameter. The fistula was flushed with the 37°C balance liquid, and the endoscope was put in the fistula to check the arachnoid at the bottom, i.e. liliequist membrane. A pair of forceps or balloon was used to penetrate the membrane to confirm the fistula was unobstructed (Fig. 1d).

A child patient with pinealoma was observed endoscopically that the tumor tissue had penetrated into the third ventricle. After burning the tumor surface with bipolar electrocoagulation, the tumor tissue was grasped by the grasping forceps and sent to the pathologic examination. The wound was flushed with the 37°C balance liquid, and the bipolar electrocoagulation was used to stop bleeding. The postoperative pathologic examination indicated that the tumor was a germinoma, and the patient was transferred into the radiotherapy department for further radiotherapy.

After the endoscopic operation, the cerebral cortex track was filled with gelatin sponge, and the CaP ceramic samples were shaped and fitted to the skull holes (Fig. 1e), with each layer of the scalp tightly sutured. The local wound was pressure dressing for 3 days after the operation.

At 1, 7 days, 3 and 6 months postoperatively, the leaking of CSF in the patients was checked carefully. The skull CT scan was applied to determine the closure and healing of the bone. The operative effect was evaluated according to the criteria shown in Table 1.

**Table 1.** The Criteria for evaluating the operative effect

Operative effect	Description
Class I	Good wound healing, no any complications
Class II	Good wound healing, slight subcutaneous hydrops
Class III	Wound dehiscence, CSF leaking, need the second operation
Class IV	Wound breakdown, severe CSF leaking and infection, possible threat to life

## Results

### Materials characterization

**Figure 2.**
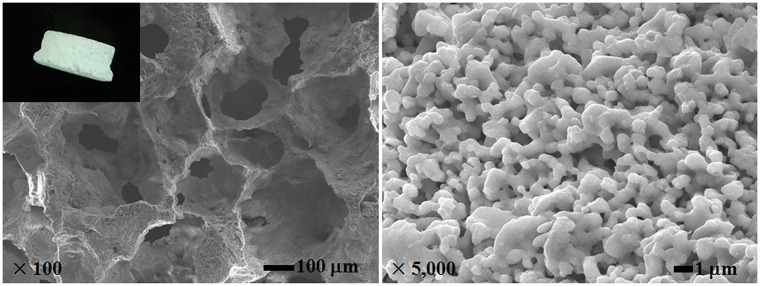
The macroscopic and microscopic morphologies of the osteoinductive CaP ceramics

Figure 2 shows the macroscopic and microscopic morphologies of the osteoinductive CaP ceramics. They had interconnected macropores (100–500 μm) and abundant micropores (<10 μm) occurred on the rough wall of those macropores. Table 2 indicates the testing result of mercury porosimetry. The porosity of this ceramic was 76.0%, and its permeability was 12.1 Darcy, showing that this ceramic could allow the penetration of body fluid under certain differential pressure.

**Table 2.** The porosity and permeability of the osteoinductive CaP ceramics

Material	Porosity, %	Permeability, Darcy
Osteoinductive CaP ceramic	76.0	12.1

### Animal experimental evaluation

**Figure 3.**
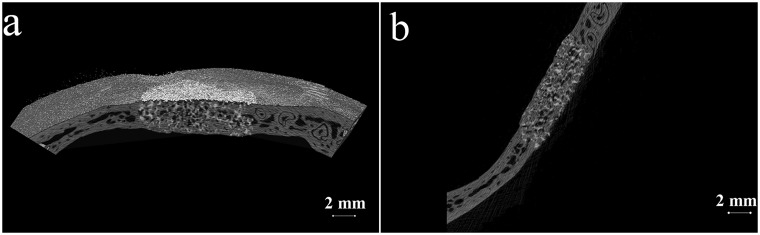
The 3D (a) and 2D (b) reconstructed images by μ-CT analysis

**Figure 4.**
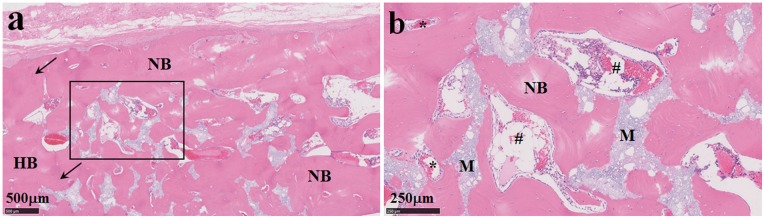
The light microscopic images of the decalcified histological sections with H&E staining (b is the partial enlarged drawing of a). Arrow, bone-implant interface; HB, host bone; NB, new bone; M, residual material; *, blood vessel; #, marrow cavity)

The postoperative gross observation indicated that the incision healed well and no macroscopic wound infection. At 6 months postoperatively, the animal was sacrificed and the retrieved implant with the surrounding skull bone was subjected to the μ-CT and histological analysis. Figure 3 shows the 3D and 2D reconstructed images. The newly formed bone at the defect site could be well distinguished from the remained material (white) by the set global grayscale threshold. The degradation of the ceramic and the bone substitution can be seen clearly. The quantitative analysis demonstrated that the newly formed bone volume density was BV/TV=65.7%. Figure 4 shows the light microscopic images of the decalcified histological sections with H&E staining. Besides some residual materials, the defect area presented a bony structure with irregular array. There were some vessels and marrow cavities formed at the defect site, in which the blood cells and myeloid tissue could be seen clearly. No obvious boundary between the implant and host bone could be observed, indicating the excellent osteointegration and bone regeneration ability of the ceramic. This result was in good accordance with the above micro-CT analysis.

### Clinical evaluation

**Figure 5.**
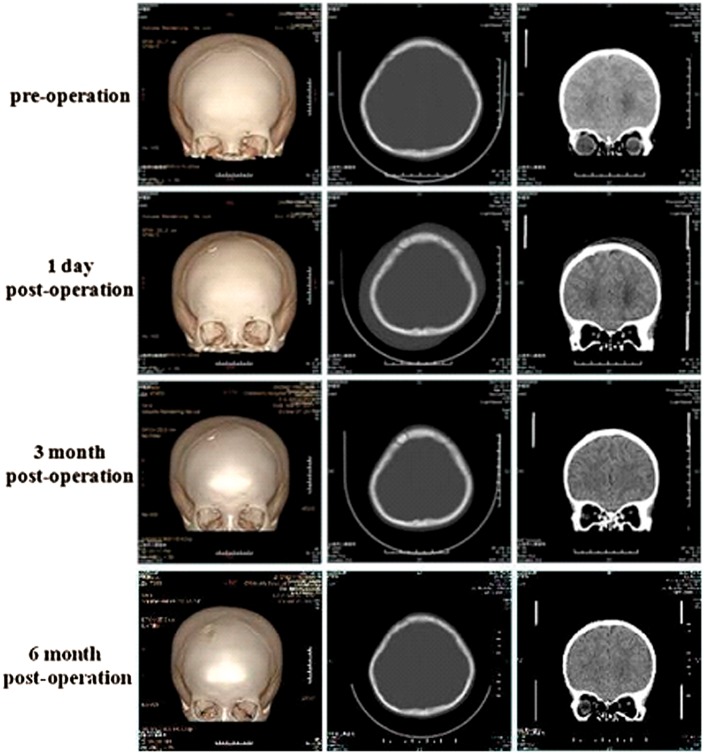
The skull CT scan before and after operation

The operations on all the five cases of children patients were carried out successfully. After follow-up for 6 months, all the five cases obtained the Class I operative effect according to the criterion (Table 1). The usage of the CaP ceramics was convenient in the operation. The ceramics could be remodeled again according to the size and shape of the drilled holes and the thickness of bone flap. The ceramics were closely connected to the surrounding bone to seal the drilled holes in an effective way without CSF leaking. Figure 5 shows the results of the skull CT scan before and after operation. At 1 day postoperatively, the follow-up CT found that the artificial bone and the surrounding bone was tightly bonded and sealed well. The density of the artificial bone was in high accordance with that of the surrounding bone on CT. No subcutaneous effusion and infection were found based on the observation at 7 days postoperatively, indicating that the wound healed well. At 3 months postoperatively, the follow-up CT demonstrated that new bone could have formed in the drilled holes and the bone healing was basically completed. At 6 month postoperatively, the follow-up CT indicated the perfect bone healing happened at the defect site, and only the trace of the ETV operation could be seen on the skull.

## Discussion

Since Mixter performed the first case of ETV in 1923, neuroendoscopy was used in the management of hydrocephalus and got a full development later on. For children patients, the reported effective rate of curing hydrocephalus by ETV was above 60% [17]. However, children with severe hydrocephalus are generally young. Many scholars thought that infants below 2-years old, especially those <1-year old, were not suitable for ETV due to the high failure rate [18–20]. The possible reason is that the absorption mechanism of CSF in infants is immature, leading to a poor absorption result. Besides, the postoperative stoma is easy to be closed again, or the new membrane could be formed. Moreover, infants with severe hydrocephalus are prone to have postoperative CSF leaking from scalp due to the severe ventricular dilatation, thin cortex and head circumference [3]. Generally, the infants patients have increasing head circumference and thinned scalp, CSF leaking from the cerebral cortex track and drilled skull holes can thus lead to the poor wound healing, wound infection and even intracranial infection [19, 21].

The common methods dealing with CSF leaking from the scalp includes the tight suture of dura, pressure dressing, subcutaneous drainage, lumbar cistern drainage etc. [22–24]. As the size of the drilled holes is only about 1 cm during the endoscopic surgery, the dura is hard to be sutured tightly. Moreover, the physiological characteristics of children who may be in a restless state after operation would influence the positive effect of the methods mentioned above. Therefore, some other treatments, such as the size and position adjustment of the drilled holes, application of bone wax sealing and so forth, were tried [7, 11]. However, in our previous clinical practice, a case of delayed healing of the scalp wound was observed, and it could be resulted from the foreign body reaction of bio-inert bone wax. Although the appearance of the patient is not affected by the minor skull holes, there are still some potential risks for infants and little children.

Thus far, besides the excellent biocompatibility and osteoconductivity, the osteoinductivity of CaP ceramics with specific porous structure and phase composition has been well confirmed by many literature reports [12, 14, 25–30]. The CaP ceramics often exhibit excellent bone healing effect when used for large bone defect repair. Yuan et al. [31] reported that the osteoinductive β-TCP ceramic had the similar repairing effect with the autologous bone graft at a critical-sized iliac bone defects. Zhu et al. [32] used a HA whisker strengthened CaP ceramic to treat the segmental femoral bone defects at a beagle model, and a mass of new bone formation at the defect sites and a high fracture load were acquired after implantation for 3 months. In this study, the osteoinductive CaP ceramics, whose angiogenesis and osteogenesis abilities has been well confirmed in our previous studies [30, 33–35], were firstly used for the repair of skull defects at a monkey model. The excellent bone regeneration and degradability were well demonstrated by the μ-CT analysis and histological evaluation (Figs. 3 and 4), indicating the great potential of the CaP ceramics in the repair of cranial defects.

According to the data shown in Fig. 2 and Table 2, the CaP ceramics had good interconnected macropores, abundant micropores on the skeleton and high porosity, endowing it with certain permeability. This meant that the porous CaP ceramics could have better air permeability, which was favorable for release of the intracranial pressure. However, only under relatively high differential pressure, the body fluid could penetrate through the porous CaP ceramics. Besides, as mentioned above, the porous CaP ceramics could promote the rapid healing of bone defects. As thus, the porous CaP ceramics could have the potential to prevent the CSF leaking. In this work, the CaP ceramics were used to treat the drilled skull holes for the first time in children’s endoscopic neurosurgery. It was found that the porous CaP ceramics had good plasticity and can be easily reshaped again in the operation, in order to adapt to the different holes drilled by surgeons. They presented good histocompatibility and well attached to the skull holes without the drifting and shifting phenomena. In the 6 months of follow-up period, all the five cases reached to the operative effect of Class I based on the evaluation criterion (Fig. 5). No CSF leaking was observed at each case. Moreover, the induced new bone formation by the ceramics and the excellent osteointegration with the surrounding bone led to the complete sealing of the skull holes, so as to further maintain the integrity of the skull of the patients. Therefore, the authors believe that the osteoinductive CaP ceramics have the great potential in treating the skull defects in children’s endoscopic neurosurgery.

## Conclusions

This study confirmed that the osteoinductive CaP ceramics could have excellent osteointegration and bone regeneration ability when used for repairing skull defect. During children’s neuroendoscopic surgery, this ceramic could be an excellent choice to deal with the drilled skull hole because it can prevent CSF leaking effectively. In addition, it is convenient to use and can lead to the rapid bone healing in the skull holes after operation. To obtain the more reliable clinical evaluation, further large cases and long-term follow-up observation are necessary.
